# Characteristics of Alcohol, Marijuana, and Other Drug Use Among Persons Aged 13–18 Years Being Assessed for Substance Use Disorder Treatment — United States, 2014–2022

**DOI:** 10.15585/mmwr.mm7305a1

**Published:** 2024-02-08

**Authors:** Sarah Connolly, Taryn Dailey Govoni, Xinyi Jiang, Andrew Terranella, Gery P. Guy, Jody L. Green, Christina Mikosz

**Affiliations:** ^1^Epidemic Intelligence Service, CDC; ^2^Division of Overdose Prevention, National Center for Injury Prevention and Control, CDC; ^3^Inflexxion, Irvine, California.

**Summary**

**What is already known about this topic?**

Substance use, including drugs and alcohol, often begins during adolescence.

**What is added by this report?**

Among adolescents being assessed for substance use disorder treatment, the most commonly reported reasons for substance use included seeking to feel mellow or calm, experimentation, and other stress-related motivations. Most reported using substances with friends; however, approximately one half of respondents who reported past–30-day prescription drug misuse reported using alone.

**What are the implications for public health practice?**

Reducing stress and promoting mental health among adolescents might lessen motivations for substance use. Educating adolescents on harm reduction practices, including the risks of using drugs alone and ensuring they are able to recognize and respond to overdose (e.g., administering naloxone), could prevent fatal overdoses.

## Abstract

Substance use often begins during adolescence, placing youths at risk for fatal overdose and substance use disorders (SUD) in adulthood. Understanding the motivations reported by adolescents for using alcohol, marijuana, and other drugs and the persons with whom they use these substances could guide strategies to prevent or reduce substance use and its related consequences among adolescents. A cross-sectional study was conducted among adolescents being assessed for SUD treatment in the United States during 2014–2022, to examine self-reported motivations for using substances and the persons with whom substances were used. The most commonly reported motivation for substance use was “to feel mellow, calm, or relaxed” (73%), with other stress-related motivations among the top reasons, including “to stop worrying about a problem or to forget bad memories” (44%) and “to help with depression or anxiety” (40%); one half (50%) reported using substances “to have fun or experiment.” The majority of adolescents reported using substances with friends (81%) or using alone (50%). These findings suggest that interventions related to reducing stress and addressing mental health concerns might reduce these leading motivations for substance use among adolescents. Education for adolescents about harm reduction strategies, including the danger of using drugs while alone and how to recognize and respond to an overdose, can reduce the risk for fatal overdose.

## Introduction

Initiation of substance use often occurs during adolescence (*1*), and adolescents commonly report using substances to feel good or get high and to relieve pain or aid with sleep problems (*2*,*3*). Adverse consequences of adolescent substance use include overdose, risk for development of substance use disorder (SUD), negative impact on brain development, and death. Prescription opioid misuse during adolescence is associated with SUD in adulthood (*4*). In the event of an overdose, immediate medical attention is necessary; bystanders can respond by calling emergency medical personnel and administering naloxone, which reverses overdoses caused by opioids. To guide the development and implementation of prevention strategies and help reduce substance use and fatal overdoses among youths, the motivations for substance use and the persons with whom adolescents report using substances were studied.

## Methods

### Data Source

Data were obtained from the National Addictions Vigilance Intervention and Prevention Program’s Comprehensive Health Assessment for Teens (CHAT) (*5*). CHAT is a self-reported, online assessment for persons aged 13–18 years who are being evaluated for SUD treatment. Assessments conducted during January 1, 2014–September 28, 2022, were analyzed. Because the assessment may be completed more than once, assessments completed by the same person within 60 days of a previous assessment were removed. The data set was restricted to assessments reporting past–30-day use of alcohol, marijuana, or other drugs* and with at least one option selected for motivation or persons with whom substances were used.

Respondents were asked to report specific substances used within six categories: 1) alcohol, 2) marijuana, hashish, or tetrahydrocannabinol (THC), 3) drugs other than alcohol or marijuana,^†^ and misuse^§^ of 4) prescription pain medications,^¶^ 5) prescription stimulants,** or 6) prescription sedatives or tranquilizers.^††^ Motivation for use was asked for each of the six categories; each motivation question had 15 response options^§§^ and respondents were asked to select all options that applied. Respondents were also asked to select the persons with whom they used substances from four categories of substances: 1) alcohol, 2) marijuana, hashish, or THC, 3) drugs other than alcohol or marijuana, and 4) prescription drugs (which included prescription pain medications, prescription stimulants, and prescription sedatives or tranquilizers). Ten options describing the persons with whom substances were used were presented,^¶¶^ and respondents were asked to select all that applied.

### Data Analysis

The percentages of each motivation and the persons with whom substances were used were calculated.*** Responses were not mutually exclusive: a respondent could report more than one motivation or person with whom substances were used; therefore, the percentages sum to >100. R software (version 4.2.2; R Foundation) was used to conduct all analyses. This activity was reviewed by CDC, deemed not research, and was conducted consistent with applicable federal law and CDC policy.^†††^

## Results

### Substance Use

Among 15,963 CHAT assessments conducted during the study period, 9,557 (60%) indicated past–30-day use of alcohol, marijuana, or other drugs. Of those, 9,543 reported at least one motivation or person with whom substances were used and were included in further analyses. Marijuana was most commonly reported (84% of assessments), followed by alcohol (49%) (Figure) (Table). Nonprescription drug use was indicated on 2,032 (21%) assessments; those most commonly reported were methamphetamine (8%), cough syrup (7%), and hallucinogens (6%). Prescription drug misuse was indicated on 1,812 (19%) assessments, with prescription pain medication reported most commonly (13%), followed by prescription sedatives or tranquilizers (11%), and prescription stimulants (9%).

**FIGURE:**
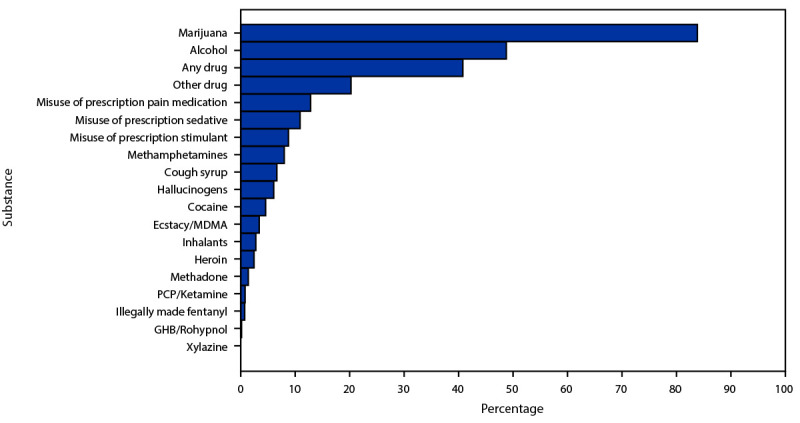
Percentage of persons aged 13–18 years being assessed for substance use disorder treatment reporting specific substances used during the previous 30 days* — National Addictions Vigilance Intervention and Prevention Program Comprehensive Health Assessment for Teens, United States, 2014–2022 **Abbreviations:** GHB = gamma hydroxybutyrate; MDMA = 3,4-methylenedioxy-methamphetamine; PCP = phenylcyclidine. * Among those reporting previous 30-day use of any alcohol, marijuana, or other drugs, and at least one motivation or person with whom substances were used.

**TABLE:** Motivations for drug use among persons aged 13–18 years being assessed for substance use disorder treatment who reported use of alcohol, marijuana, or other drugs during the previous 30 days and persons with whom they used substances — National Addictions Vigilance Intervention and Prevention Program Comprehensive Health Assessment for Teens, United States, 2014–2022

Measure	No. (%)							
	Overall* 9,543 (100)	Alcohol^†^ 4,648 (49)	Marijuana^§,¶^ 7,994 (84)	Nonprescription drug^¶^ 2,032 (21)	Prescription medication			
					Pain medication** 1,222 (13)	Stimulant^††^ 834 (9)	Sedative/ Tranquilizer^§§^ 1,037 (11)	Any^¶¶^
Motivation***								
To feel mellow, calm, or relaxed	**6,968 (73)**	1,862 (40)	6,090 (76)	1,085 (53)	745 (61)	243 (29)	569 (55)	—
To sleep better or fall asleep	**4,216 (44)**	620 (13)	3,644 (46)	560 (28)	425 (35)	94 (11)	364 (35)	—
To stay awake	**1,212 (13)**	133 (3)	309 (4)	618 (30)	128 (10)	262 (31)	66 (6)	—
To feel less shy or more social	**2,056 (22)**	926 (20)	1,183 (15)	456 (22)	152 (12)	111 (13)	116 (11)	—
To stop worrying about a problem or forget bad memories	**4,169 (44)**	1,514 (33)	3,148 (39)	869 (43)	382 (31)	165 (20)	276 (27)	—
To have fun or experiment	**4,771 (50)**	2,372 (51)	3,157 (39)	1,124 (55)	431 (35)	248 (30)	330 (32)	—
To be sexier or make sex more fun	**1,033 (11)**	441 (10)	664 (8)	320 (16)	107 (9)	51 (6)	52 (5)	—
To lose weight	**400 (4)**	46 (1)	104 (1)	199 (10)	40 (3)	54 (7)	20 (2)	—
To make something less boring	**3,893 (41)**	1,634 (35)	2,846 (36)	895 (44)	361 (30)	221 (26)	259 (25)	—
To improve or get rid of the effects of other drugs	**1,008 (11)**	356 (8)	640 (8)	393 (19)	183 (15)	101 (12)	132 (13)	—
To concentrate better	**2,126 (22)**	84 (2)	1,637 (20)	412 (20)	121 (10)	230 (28)	74 (7)	—
To deal with chronic pain	**1,326 (14)**	121 (3)	1,055 (13)	227 (11)	231 (19)	44 (5)	80 (8)	—
To help with depression or anxiety	**3,787 (40)**	1,087 (23)	3,068 (38)	840 (41)	398 (33)	191 (23)	328 (32)	—
To fit in	**1,144 (12)**	487 (10)	641 (8)	226 (11)	87 (7)	49 (6)	49 (5)	—
Other reason	**2,149 (23)**	704 (15)	1,074 (13)	318 (16)	176 (14)	120 (14)	133 (13)	—
Median no. of motivations selected*** (IQR)	**3 (2–6)**	2 (1–4)	3 (2–5)	3 (1–6)	2 (1–5)	2 (1–4)	2 (1–4)	—
**Persons with whom substances were used**^¶¶,^***								
Friend or friends	**7,751 (81)**	3,906 (84)	6,419 (80)	1,581 (78)	—	—	—	1,168 (64)
Brother or sister	**1,273 (13)**	427 (9)	1,018 (13)	128 (6)	—	—	—	55 (3)
Parent or parents	**389 (4)**	187 (4)	195 (2)	52 (3)	—	—	—	29 (2)
Adult relative or other adult	**881 (9)**	375 (8)	591 (7)	156 (8)	—	—	—	78 (4)
Relative near your own age	**865 (9)**	288 (6)	662 (8)	98 (5)	—	—	—	45 (3)
Boyfriend or girlfriend	**2,288 (24)**	1,066 (23)	1,771 (22)	449 (22)	—	—	—	256 (14)
Coworker	**302 (3)**	88 (2)	252 (3)	45 (2)	—	—	—	20 (1)
Someone else	**1,610 (17)**	507 (11)	1,135 (14)	368 (18)	—	—	—	173 (10)
Anyone who has drugs	**2,189 (23)**	767 (17)	1,762 (22)	472 (23)	—	—	—	284 (16)
Alone	**4,757 (50)**	1,200 (26)	3,526 (44)	798 (39)	—	—	—	931 (51)
Median no. of persons with whom substances were used*** (IQR)	**2 (1–3)**	1 (1–2)	2 (1–3)	2 (1–3)	—	—	—	1 (1–2)

**Abbreviation:** THC = tetrahydrocannabinol.

* Includes motivations or persons with whom adolescents used substances reported for any of the following: alcohol, marijuana, nonprescription drugs, prescription drug misuse, methadone, “other drug,” and “any drug.”

**^†^** The alcohol motivation question is phrased, “People use alcohol for many reasons. Why have you used alcohol? Select all that apply.” The question asking with whom alcohol is used is phrased, “When you drink, who do you drink with? Select all that apply.”

**^§^** The marijuana motivation question is phrased, “People use marijuana, hashish, or THC for many reasons. Why have you used marijuana, hashish, or THC? Select all that apply.” The question asking with whom marijuana is used is phrased, “When you use marijuana, hashish, or THC, who do you use it with? Select all that apply.”

^¶^ Inhalants, cocaine, methamphetamines, hallucinogens, phenylcyclidine or ketamine, heroin, ecstasy or 3,4-methylenedioxy-methamphetamine, gamma hydroxybutyrate or rohypnol, cough syrup, illegally made fentanyl (added to assessment in 2017), and xylazine (added to assessment in 2022). The motivation question is phrased, “People use drugs for many reasons. Why have you used drugs, other than alcohol or marijuana? Select all that apply.” The question asking with whom these substances are used is phrased, “When you use drugs, other than alcohol or marijuana, who do you use them with? Select all that apply.” This assessment section also included methadone, “other drug,” and “any drug,” which are captured by the same motivation question and the question asking with whom persons use. If a person reported methadone, “other drug,” or “any drug” in addition to one or more nonprescription drugs, the motivations and with whom they use (for methadone, “other drug,” or “any drug”) cannot be differentiated and are counted in this table.

** Includes persons who responded affirmatively to assessment questions asking about prescription pain medication use “not as prescribed,” “without a prescription from a doctor,” “to get high,” or “to change how you feel.” The motivation question is phrased, “People use drugs for many reasons. Why have you used prescription pain medications on your own? Select all that apply.”

^††^ Includes persons who responded affirmatively to assessment questions asking about prescription stimulant use “not as prescribed,” “without a prescription from a doctor,” “to get high,” or “to change how you feel.” The motivation question is phrased, “People use drugs for many reasons. Why have you used prescription stimulants on your own? Select all that apply.”

^§§^ Includes persons who responded affirmatively to assessment questions asking about prescription sedative and tranquilizer use “not as prescribed,” “without a prescription from a doctor,” “to get high,” or “to change how you feel.” The motivation question is phrased, “People use drugs for many reasons. Why have you used prescription sedatives or tranquilizers on your own? Select all that apply.”

^¶¶^ The question asking with whom substances are used is asked once for all prescription drugs and is phrased, “When you use prescription drugs, who do you use them with? Select all that apply.” The denominator for the number of assessments indicating past–30-day misuse of at least one prescription drug is 1,812.

*** Motivation and persons with whom substances are used questions are in a “select all that apply” format; therefore, percentages sum to >100. Median and IQR summarize the number of motivations and the number of persons with whom they use substances that respondents selected for each question.

### Reasons Reported for Using Substances

Overall, the most common reasons adolescents reported for using substances were to feel mellow, calm, or relaxed (73%), to have fun or experiment (50%), to sleep better or to fall asleep (44%), to stop worrying about a problem or to forget bad memories (44%), to make something less boring (41%), and to help with depression or anxiety (40%). By category, the most frequently reported motivation for alcohol use and nonprescription drug misuse was to have fun or experiment (51% and 55%, respectively), whereas use to feel mellow, calm, or relaxed was the most reported motivation for use of marijuana (76%), and misuse of prescription pain medications (61%) and prescription sedatives or tranquilizers (55%). The most common motivation for prescription stimulant misuse was to stay awake (31%).

### Persons with Whom Substances Were Used

Adolescents most commonly used substances with friends (81%), a boyfriend or girlfriend (24%), anyone who has drugs (23%), and someone else (17%); however, one half (50%) reported using alone. Although using with friends and using alone were reported most often for all substances, the prevalence varied by substance type. Approximately 80% of adolescents who reported using alcohol, marijuana, or nonprescription drugs reported using these substances with friends; however, 64% of those who reported misusing prescription drugs used them with friends. Among adolescents reporting prescription drug misuse, more than one half (51%) reported using these drugs alone, whereas using alone was reported by 44% of those who used marijuana, 39% of those who used nonprescription drugs, and 26% of those who used alcohol.

## Discussion

This analysis summarizing self-reported motivations for use of various substances among adolescents being assessed for SUD treatment who used alcohol, marijuana, or other drugs during the previous 30 days, and the persons with whom adolescents used these substances, found that many adolescents use substances to have fun or experiment or to seek relief mentally, emotionally, or physically. These findings are consistent with those reported in a 2020 study that examined motivations for the nonmedical use of prescription drugs in a sample of young adults, which identified recreational and self-treatment motivations among young adults over time and across drug classes (*2*). Anxiety and experiencing traumatic life events have been associated with substance use in adolescents (*6*). Specific reporting of motivations, including “to stop worrying about a problem or to forget bad memories” and “to help with depression or anxiety,” underscores the potential direct impact that improving mental health could have on substance use.

One half of adolescents reported using substances while alone. Of particular concern, more than one half of respondents who reported past–30-day prescription drug misuse reported using the drugs alone. Prescription drug misuse while alone presents a significant risk for fatal overdose, especially given the proliferation of counterfeit pills resembling prescription drugs and containing illegal drugs (e.g., illegally manufactured fentanyl) (*7*). Education about harm reduction behaviors, such as using in the presence of others and expanding access to naloxone to all persons who use drugs, could reduce this risk.

Adolescents most commonly reported using substances with friends, which presents the opportunity for bystander intervention in the event of an overdose. Nearly 70% of fatal adolescent overdoses occurred with a potential bystander present, yet in most cases no bystander response was documented (*8*). Overdose deaths can be prevented through education tailored to adolescents to improve recognition of signs of overdose and teach bystanders how to respond, including the administration of naloxone (*9*) and increasing awareness of local Good Samaritan laws, which protect persons against liability when they provide emergency care to others (*10*). In addition, ensuring access to effective, evidence-based treatment for SUD and mental health conditions might decrease overdose risk.

### Limitations

The findings in this report are subject to at least three limitations. First, the population represents a convenience sample of adolescents being assessed for SUD treatment and is not generalizable to all adolescents in the United States. Second, the assessment is self-reported and subject to potential reporting and recall biases as well as social desirability bias. Finally, several questions on motivations and persons with whom respondents use substances refer to categories of substances; thus, it was not possible to ascertain to which specific drug a person might be referring in their response if use of more than one substance within a drug category was reported.

### Implications for Public Health Practice

Harm reduction education specifically tailored to adolescents has the potential to discourage using substances while alone and teach how to recognize and respond to an overdose in others, which could thereby prevent overdoses that occur when adolescents use drugs with friends from becoming fatal. Public health action ensuring that youths have access to treatment and support for mental health concerns and stress could reduce some of the reported motivations for substance use. These interventions could be implemented on a broad or local scale to improve adolescent well-being and reduce harms related to substance use.

## Footnotes

* Two assessments that reported using only methadone were excluded.

† The category “drugs, other than alcohol or marijuana” included the following nonprescription drugs: inhalants, cocaine, methamphetamines, hallucinogens, phenylcyclidine or ketamine, heroin, ecstasy or 3,4-methylenedioxy-methamphetamine, gamma hydroxybutyrate or rohypnol, cough syrup, illegally made fentanyl (added to assessment in 2017), and xylazine (added to assessment in 2022), methadone, “other drug,” and “any drug.”

§ Misuse is described as prescription medication use “not as prescribed,” “without a prescription from a doctor,” “to get high,” or “to change how you feel.”

¶ A description of prescription pain medications provided in the assessment states, “Examples of painkillers include Oxycontin, Vicodin, and Percocet. Pain medications help people feel less pain after surgery, and help manage intense chronic pain.”

** A description of prescription stimulants provided in the assessment states, “Examples of stimulants include Ritalin, Adderall, and Dexedrine. Stimulants help people concentrate or focus better.”

†† A description of prescription sedatives or tranquilizers provided in the assessment states, “Examples of sedatives include Valium, Xanax, and Klonopin. Sedatives or tranquilizers help people sleep or feel less anxious.”

§§ 1) To feel mellow, calm, or relaxed, 2) to sleep better or fall asleep, 3) to stay awake, 4) to feel less shy or more social, 5) to stop worrying about a problem or forget bad memories, 6) to have fun or experiment, 7) to be sexier or make sex more fun, 8) to lose weight, 9) to make something less boring, 10) to improve or get rid of the effects of other drugs, 11) to concentrate better, 12) to deal with chronic pain, 13) to help with depression or anxiety, 14) to fit in, or 15) other reasons.

¶¶ 1) Friend or friends, 2) brother or sister, 3) parent or parents, 4) adult relative or other adult, 5) relative near adolescent’s own age, 6) boyfriend or girlfriend, 7) coworker, 8) someone else, 9) anyone who has drugs, or 10) used alone.

*** The number of assessments for which an option was selected was divided by the total number of assessments in that substance type category.

††† 45 C.F.R. part 46.102(l)(2), 21 C.F.R. part 56; 42 U.S.C. Sect. 241(d); 5 U.S.C. Sect. 552a; 44 U.S.C. Sect. 3501 et seq.
